# Chemoresistive Gas Sensors for the Detection of Colorectal Cancer Biomarkers

**DOI:** 10.3390/s141018982

**Published:** 2014-10-13

**Authors:** Cesare Malagù, Barbara Fabbri, Sandro Gherardi, Alessio Giberti, Vincenzo Guidi, Nicolò Landini, Giulia Zonta

**Affiliations:** 1 Department of Physics and Earth Science, University of Ferrara, Via Saragat 1/c, Ferrara 44122, Italy; E-Mails: fbbbbr@unife.it (B.F.); gherardi@fe.infn.it (S.G.); giberti@fe.infn.it (A.G.); guidi@fe.infn.it (V.G.); nicolo.landini@student.unife.it (N.L.); giulia.zonta@unife.it (G.Z.); 2 CNR-INO—Istituto Nazionale di Ottica, Largo Enrico Fermi 6, Firenze 50124, Italy; 3 MIST E-R s.c.r.l., Via P. Gobetti 101, Bologna 40129, Italy

**Keywords:** gas, sensors, nanotechnology, semiconductors, metal oxides, colorectal cancer, VOC, 1-iodo-nonane

## Abstract

Numerous medical studies show that tumor growth is accompanied by protein changes that may lead to the peroxidation of the cell membrane with consequent emission of volatile organic compounds (VOCs) by breath or intestinal gases that should be seen as biomarkers for colorectal cancer (CRC). The analysis of VOCs represents a non-invasive and potentially inexpensive preliminary screening technique. An array of chemoresistive gas sensors based on screen-printed metal oxide semiconducting films has been selected to discriminate gases of oncological interest, e.g., 1-iodononane and benzene, widely assumed to be biomarkers of colorectal cancer, from those of interference in the gut, such as methane and nitric oxide.

## 1. Introduction

Nanotechnology represents a promising means for detection, staging and treatment of cancer [1,2]. Colorectal cancer (CRC) is the third most diffused form of cancer in the world, with nearly 1.4 million new cases diagnosed in 2012. The American Cancer Society [3] estimates that in 2011, about 141,210 people in the USA will be diagnosed with CRC and about 49,380 people will die because of it. If diagnosed early, CRC is also one of the most curable types of cancer, with cure rates as high as 90%. It is more common in developed countries and mainly affects people older than 50 years. Current screening techniques, such as colonoscopy, are invasive, traumatic and unpleasant for already debilitated patients [4]. The combination of nanotechnology with the need of medicine to find a non-invasive method to detect and prevent cancer is a rapidly evolving field. It is known that VOC emissions are linked to tumor growth, which is accompanied by gene and/or protein changes that may lead to these volatile emissions [5,6]. VOCs can be considered as biomarkers for the different types of cancers, and their analysis is a new frontier in medical diagnostics, because it is non-invasive and potentially inexpensive [7–9]. Tumor VOCs can be detected directly from the headspace of cancer cells or through exhaled breath; in fact, changes in the blood chemistry lead to measurable modifications in the breath by exchanges through the lung, e.g., in the range between 20 and 100 ppb for several VOCs [10]. The aim of this work is to study the detection of VOCs as indicators of CRC, identifying the most selective sensors of these compounds. The application goal of this study is to establish a method to analyze flatulence and intestinal gases, in which it is reasonable to think that these biomarkers should be conveyed.

Concentrations of tumor VOCs in flatus are not specified in literature, so we assume that they are of the same order of magnitude of the concentrations in breath (tenths of ppb).

The most relevant VOCs that may indicate CRC are benzene compounds [10,11] and the molecule, 1-iodo-nonane (C_9_H_19_I) [10], while the most significant interfering gases are H_2_, N_2_ and NO_x_, CO_2_, CH_4_ and a small quantity of sulfur compounds, principally produced by the fermentation and digestion processes [12–15].

In this study, the idea is to find the most selective chemoresistive sensors to benzene and 1-iodo-nonane, analyzing their responses (relative conductance ratios) to these markers and to some of the principal interfering gases in the gut (as H_2_, CH_4_). In our work, we have named as “principal” interferers those gases that can reach a 10% or more of total gaseous composition inside the intestine. NO is also tested, because it is produced in the gut by intrinsic intestinal tissues, resident and/or infiltrating leukocytes, reduction of luminal gastric nitrate and denitrification by commensal anaerobes [16].

## 2. Experimental Section

A set of twelve metal-oxide semiconducting films has been selected for the purpose. The materials chosen are five solid solutions of SnO_2_ and TiO_2_ (ST20 650, ST25 650, ST25 + Au1%, ST30 650, ST50 650), a solid solution of WO_3_ and SnO_2_ (WS30), a solution of TiO_2_, Ta_2_O_5_ and vanadium oxide (TiTaV) and, finally, a solution of SnO_2_, TiO_2_ and Nb_2_TiO_7_ (STN). For a better understanding, the metal oxide composition of sensors is reported in Table 1.

**Figure 1.**
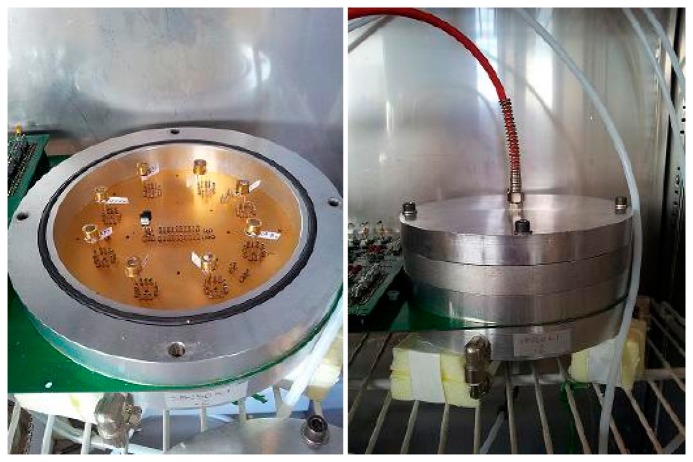
Sensors positioned inside the open chamber (**Left**) and the chamber hermetically sealed (**Right**).

Generally, the presence of SnO_2_ makes the active material sensitive to a wide range of gases, so the addition of other oxides is fundamental to refine the selectivity of the sensors. Functional materials were prepared by the sol-gel technique [17], then fired at the temperatures indicated at the end of their names and used to screen-print sensing layers onto miniaturized alumina substrates [18]. Afterwards, they were characterized with the X-ray diffraction technique (XRD), thermo-gravimetry/differential thermal analysis (TG/DTA) and scanning electron microscopy (SEM). Details about the synthesis of these materials and the deposition technique have been reported in previous works of our group [19–22]. Sensors were positioned inside a sealed test chamber (Figure 1), and conductance measurements were performed with the so-called “flow-through technique” [23,24]. The flow-rate is measured in sccm (standard cubic centimeters per minute), and it affects the rate of the surface reactions between the gas and the surface of the sensitive material.

**Figure 2.**
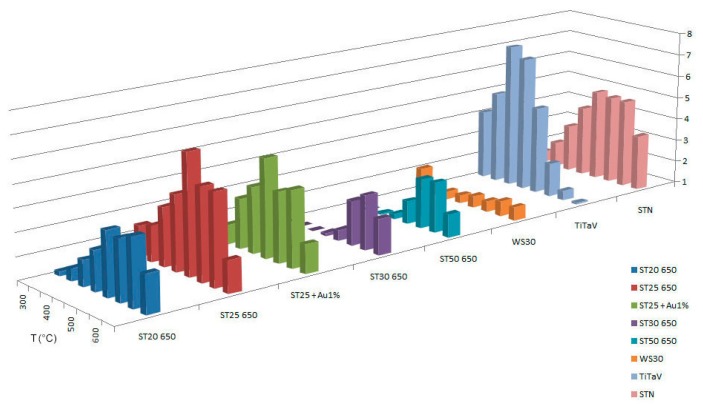
Response of sensors to C_6_H_6_ at temperatures ranging from 300 °C to 650 °C.

**Figure 3.**
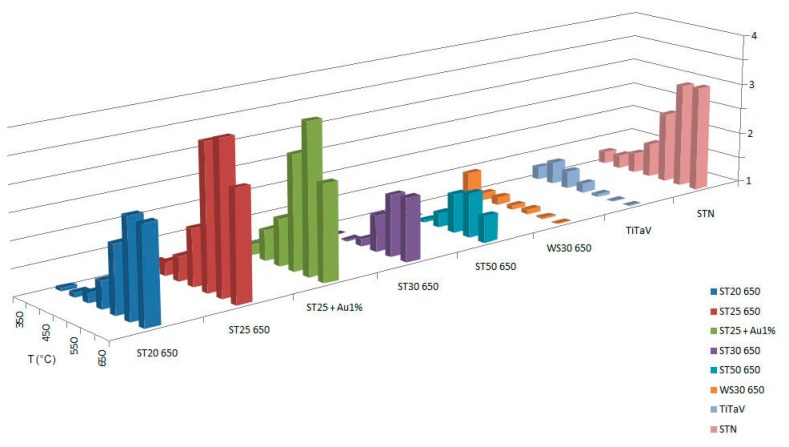
Response of sensors to CH_4_ at temperatures ranging from 350 °C to 650 °C.

**Figure 4.**
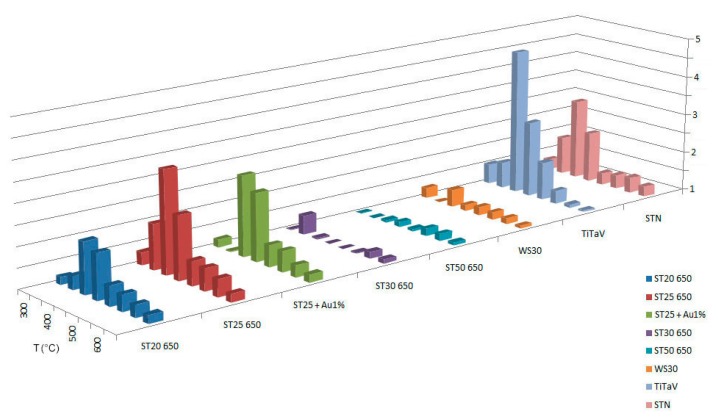
Response of sensors to NO at temperatures ranging from 300°C to 650°C.

To identify the best detecting temperature for each type of sensing material, we tested the response at several working temperatures (300 °C, 350 °C, 400 °C, 450 °C, 500 °C, 550 °C, 600 °C, 650°C). Temperatures lower than 300°C are not taken into account, because, at low temperatures, the metal-oxide sensors used in this work do not show a stable response. The working temperatures are set by applying an external voltage *V_h_* to the heating circuit of each sensor, whose resistance is indicated here with *R_h_*. Therefore, it is possible to control and directly modify *R_h_*, which determines the sensor's temperature. The temperature of the chamber (36–37°C) is directly influenced by the sensors working temperatures and remains almost constant. We performed this temperature analysis in dry conditions (synthetic dry air with 20% of O_2_ and 80% of N_2_) with the following target gases: C_6_H_6_ (2 ppm), CH_4_ (50 ppm), NO (5 ppm), and we made some interfering tests also with H_2_ (60 ppm) and humidity. CO_2_ is not considered, because it is well known that it is hardly detected by chemoresistive sensors [19]. For methane T=300°C was not considered, because is too low for sensors to generate a response. Dry conditions were chosen to show the absolute response to the gases of interest; in fact, even if humidity is present in our intestine, generally diminishing the sensors response, it does not conspicuously change the response ratios between benzene and its interferers. Measurements in wet conditions are in progress and not presented here. The results are summarized in Figures 2, 3 and 4.

The concentrations chosen for interferers are based on the fact that we want to be able to detect benzene even in the unfortunate case that the gut is filled with fermentation products. Therefore, CH_4_ and NO are tested with a greater concentration than that of benzene. On the other hand, when a gastrointestinal exam occurs, normally, the patient has to take a particular diet some days before the test, in order to reduce the amount of disturbing gaseous compounds inside the intestine. Then, some interference tests were made. We tested C_6_H_6_ + CH_4_ at the best temperature for benzene, derived from the previous analysis.

After selecting the most sensitive materials for benzene, we tested them in dry conditions with C_6_H_6_ + H_2_ (2, 60 ppm) C_6_H_6_ + H_2_ + CH_4_ (2, 60, 10 ppm) and C_6_H_6_ + H_2_ + CH_4_ (2, 60, 10 ppm) using humidity as an interferer (RH = 37%).

**Figure 5.**
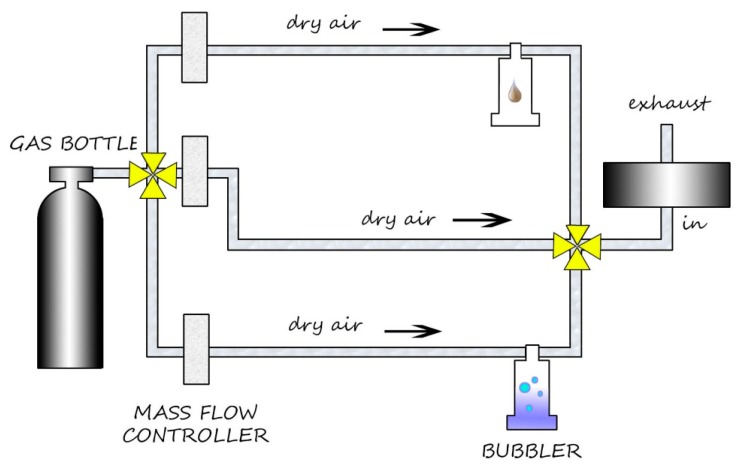
Measurement apparatus for 1-iodo-nonane: two-bubbler system.

**Figure 6.**
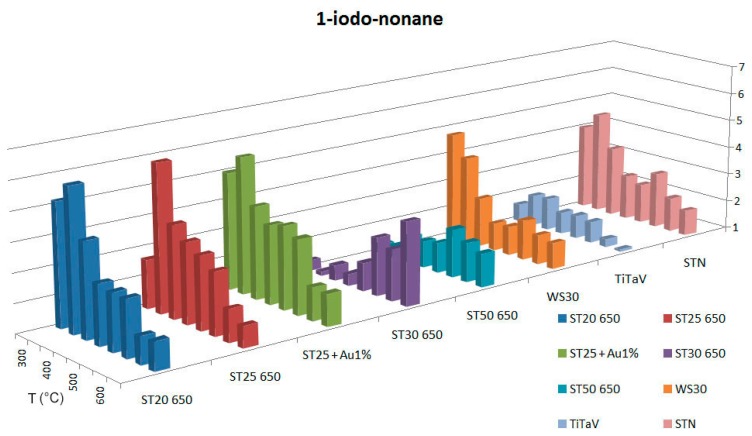
Responses of sensors to 1-iodo-nonane for temperatures in the interval between 300 °C and 650 °C.

In the second part, the same analysis is performed with the VOC, 1-iodo-nonane (chemical formula: C_9_H_19_I [25]), with the apparatus shown in Figure 5. We obtained the responses as a function of the temperature shown in Figure 6.

Tests were done in wet conditions (with a constant relative humidity (RH) ∼18% inside the volume of the chamber) to reproduce the intestinal environment. A fixed fraction of the total flux came from the gas bubbler filled with distilled water, while the remaining fraction was composed of two lines, one of synthetic dry air and the other passing through a second gas bubbler in which there were some drops of 1-iodononane. After the stabilization of sensors in wet air, a drop of 1-iodo-nonane is put inside the gas bubbler after being weighed with a precision balance (accuracy of 10^−5^g). After the measurement, the concentration has been calculated, dividing the quantity of 1-iodo-nonane, just measured before, by the evaporation time, taking into account also the volume of 1-iodo-nonane in the chamber and the volume of the chamber itself. The characteristics of 1-iodo-nonane used for the tests are listed in Table 2.

## 3. Results and Discussion

From the experimental data presented in the section above, it can be observed that the sensors that are most sensitive to benzene are, in order of decreasing response, R (defined as the ratio of the conductance in gas and the conductance in air): TiTaV (peak of 7.43 at 400°C), ST25 650 (peak of 6.22 at 500°C), ST25 + Au1% (peak of 5.28 at 500°C), STN (peak of 5.04 at 500°C, 4.95 at 550°C and 4.91 at 600°C) and ST20 650 (peak of 3.93 at 600°C). For methane, the most sensitive sensors are: ST25 650 (peak of 3.85 at 600°C), ST25 + Au1% (peak of 3.85 at 600°C) and STN (peak of 3.06 at 650°C and of 3.04 at 600°C). We determined from these observations that the best sensors to detect benzene tend to be the same as those to detect methane. However, it is possible to choose, for each sensor, a specific temperature at which it is more selective to benzene than to methane (*T_best_*): These temperatures are listed in Table 3.

**Figure 7.**
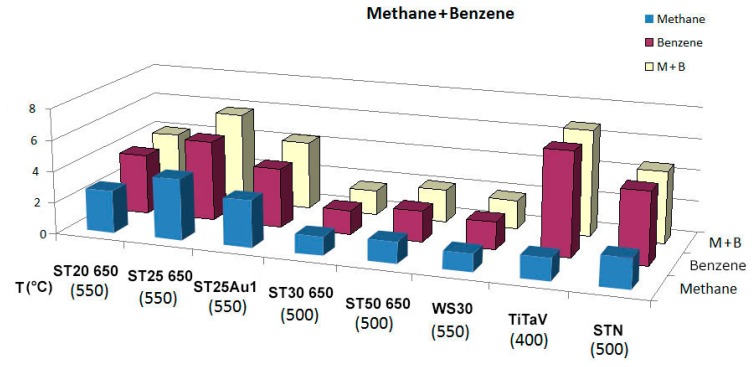
Bar-chart of the response of the sensors to CH_4_, C_6_H_6_ and to the two gases in combination at *T_best_*.

In order to test the interference of methane in the detection of benzene, the two gases were injected together into the test chamber. In Figure 7 the response of the sensors to the two gases, injected separately and in combination, are reported. In particular, the response of TiTaV to C_6_H_6_ is 4.76-times higher than that to CH_4_, and the response of STN to C_6_H_6_ is 1.31-times higher than that to CH_4_, at these concentrations.

The NO tests resulted in a relatively peaked temperature dependence; therefore, it is possible to tune the temperature to avoid the effects of the undesired compound. As we can see from the experimental data, the most selective sensors for benzene with respect to NO are ST25 650 at 500°C (ratio 3.91), and STN at 500°C (ratio 3.88).

**Figure 8.**
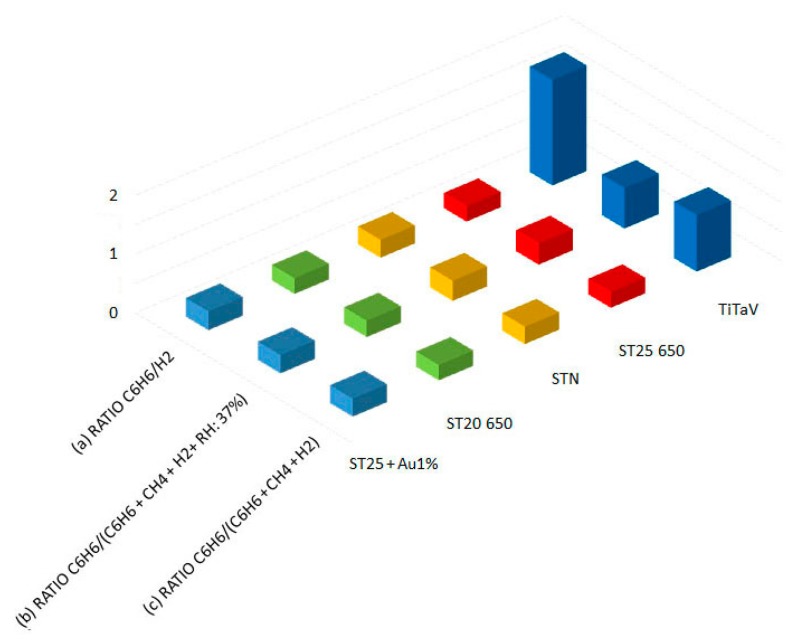
Responses in dry conditions of some of the most sensitive sensors to benzene to the following mixtures: (**a**) C_6_H_6_ + H_2_ (2, 60 ppm); (**b**) C_6_H_6_ + H_2_ + CH_4_ (2, 60, 10 ppm); and (**c**) C_6_H_6_ + H_2_ + CH_4_ (2, 60, 10 ppm) using humidity as the interferer (RH = 37%).

As anticipated in Section 2, Figure 8 shows the ratios between benzene and interferers as methane, hydrogen and water vapor. From Line (a), we see that the responses of ST25 650, ST20 650, STN and ST25 + Au1% are considerably affected by the presence of H_2_, while the response of TiTaV remains almost unaffected (the ratio C_6_H_6_/H_2_ is 1.8). Comparing Lines (b) and (c), it is evident that humidity tends to reduce the response of TiTaV in the presence of H_2_ and CH_4_; however, it remains a selective material for benzene (C_6_H_6_/(C_6_H_6_ + CH_4_ + H_2_) ∼1 without humidity and ∼0.7 with RH = 37%).

Tests with 1-iodo-nonane were performed in a temperature range spanning from 300°C to 650°C, with steps of 50°C. The concentrations calculated for this VOC are in the range between ∼3 and 5 ppm. As reported in Figure 6, sensors with the best response to 1-iodo-nonane are, in order of decreasing response, ST25 650 (peak at 350°C of 6.05), ST20 650 (peak at 350°C of 5.87), ST25 + Au1% (peak at 350°C of 5.69), WS30 (peak at 300°C of 4.76) and STN (peak at 350°C of 4.62). The effect of NO and CH_4_ as interfering gases in the measurements of 1-iodo-nonane has been tested, resulting in a negligible disturbance.

**Figure 9.**
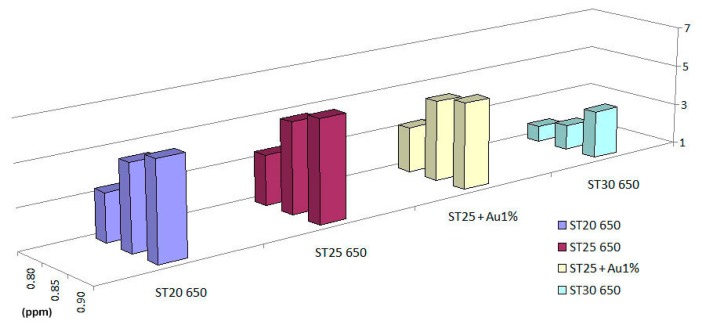
Bar-chart of the responses of sensors to 1-iodo-nonane in wet conditions (RH: 17%–19%) for the following flows: (2.024 ± 0.002) × 106 mol/min (I), (2.153 ± 0.002) × 106 mol/min and (2.271 ± 0.001) × 106 mol/min (III); these correspond to concentrations of: 0.80 (I), 0.85 (II) and 0.90 (III) ppm.

Concentration tests for 1-iodo-nonane were made with an array of sensors composed of ST20 650, ST25 650, ST25 + Au1% and ST30 650, to study how the responses vary along with concentration. RH has been fixed at about 18% during the measurement. Results are summarized in Figure 9.

The response and recovery times vary from sensor to sensor and are linked to numerous variables (e.g., working temperature of the sensors, presence of humidity, gas species and concentration). In our tests, the response time is always faster than the recovery, and these vary from a few minutes to about one hour.

## 4. Conclusions

**Figure 10.**
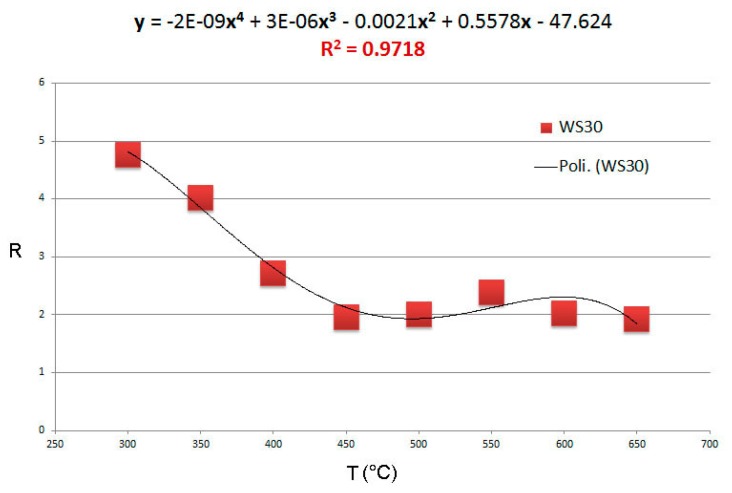
Fit for the response as a function of the temperature of WS30 650 tested with 1-iodo-nonane. The equation of the approximated polynomial (Poli.) is shown in the graphic.

A set of sensors made of single and mixed oxides has been tested with benzene and 1-iodo-nonane. An array of sensors was then obtained combining different materials working at their best temperatures, which proved to be highly selective to these gases, also in a background of realistic concentrations of CH_4_, NO and H_2_. All sensors in the array show a response that can be fitted with a fourth order polynomial with a peak around the best working temperature (an example of fit for WS30 650 in Figure 10).

The best working temperature has been determined for each sensor, and the responses of the different films to the target compounds have been analyzed. The sensors that resulted in being the most selective to benzene in dry conditions in a methane background are TiTaV (tin, tantalum and vanadium oxides) and STN (mixed tin, titanium and niobium oxides) and in NO background are ST25 650 (tin, titanium) and STN. TiTaV shows a great selectivity to benzene also when a high concentration of hydrogen is injected. Humidity tends to lower the responses without substantially changing their trends. The best sensors to detect 1-iodo-nonane in a wet ambient environment are ST20 650, ST25 650 and ST25 + Au1%, and their responses tend to increase along with concentration (order of 10^−2^ ppm). The chosen array will serve as the basis to set up an electronic network of sensors to be trained for target gases in CRC screening. This device may represent a non-invasive and potentially inexpensive pre-screening method for the diagnosis of CRC and a substitute of preventive colonoscopy.

## Figures and Tables

**Table 1.** Composition of the sensor films.

**Name**	**Film Composition**
ST20 650	SnO_2_, TiO_2_ (20%)
ST25 650	SnO_2_, TiO_2_ (25%)
ST25 + Au1%	SnO_2_, TiO_2_ (25%), Au (1%)
ST30 650	SnO_2_, TiO_2_ (30%)
ST50 650	SnO_2_, TiO_2_ (50%)
WS30	WO_3_, SnO_2_ (30%)
TiTaV	TiO_2_, Ta_2_O_5_, vanadium oxide
STN	SnO_2_, TiO_2_, Nb_2_TiO_7_

**Table 2.** Principal features of C_9_H_19_I.

**1-iodo-nonane**	
**ASSAY**	95%
**CONTAINS**	copper as stabilizer
**REFRACTIVE INDEX**	n20/D 1.487
**BOILING POINT**	107–108 °C/8 mmHg
**DENSITY**	1.288 g/mL at 25 °C
**PACKAGING**	25 g in glass bottle

**Table 3.** *T_best_* of sensors for benzene in a methane background.

**Code**	139A/A4	157A/B5	158A/A5	143A/A4	147A/A10	145A/B9	104/B9	129/D1
**Sensor**	ST20 650	ST25 650	ST25Au1	ST30 650	ST50 650	WS30	TiTaV	STN
***T****_best_*	550	550	550	500	500	550	400	500
